# Laser speckle flowgraphy findings in focal scleral nodule

**DOI:** 10.1007/s00417-021-05391-x

**Published:** 2021-09-08

**Authors:** Yui Yamashita, Michiyuki Saito, Kiriko Hirooka, Susumu Ishida

**Affiliations:** Department of Ophthalmology, Faculty of Medicine and Graduate School of Medicine, Hokkaido University, N-15, W-7, Kita-ku, Sapporo, 060-8638 Japan


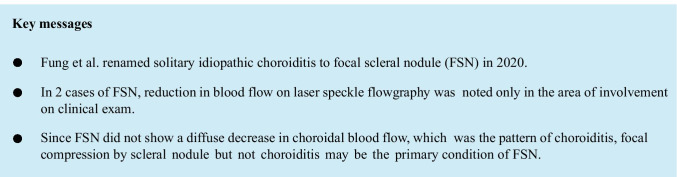


Dear Editor.

Focal scleral nodule (FSN), renamed by Fung et al. in 2020 [1], is characterized by the partial elevation of the sclera and a solitary yellowish-white spot with clear boundaries. In the FSN lesion, the choroidal circulation may be locally impaired given previously reported hypofluorescence on indocyanine green angiography (ICGA) and nonperfusion of choriocapillaris on optical coherence tomography angiography [1]. To date, surveying macular choroidal blood flow using laser speckle flowgraphy (LSFG) has played an important role in elucidating the pathophysiology of various fundus diseases [2–8]; however, the LSFG findings of FSN has not been reported so far. Here, we report the LSFG findings in 2 FSN cases.

We would like to describe 2 cases. Our first case was a 56-year-old woman who was found to have an abnormality in her right fundus. Her medical and family history was unremarkable. The patient’s best-corrected visual acuity (BCVA) was 20/20 OD. Funduscopic examination revealed an orange lesion of less than two-disc diameter on the inferonasal site of the macula (Fig. 1a). Fluorescein angiography (FA) showed scattered hypofluorescence and surrounding hyperfluorescence (Fig. 1c) in the early phase followed by granular enhancement (Fig. 1d). ICGA showed hypofluorescence in the early phase (Fig. 1e) and surrounding slight hyperfluorescence in the late phase (Fig. 1f). On enhanced depth imaging optical coherence tomography (EDI-OCT), the sclera was elevated with the overlying choroid thinned to 20 μm (Fig. 1g), whereas the central choroidal thickness was 144 μm. B-mode echography showed no acoustic shadow (Fig. 1h). Contrast-enhanced MRI of the head and orbit (Fig. 1i), gallium scintigraphy, and blood tests showed no abnormal systemic or ocular findings. The patient was diagnosed with FSN and followed up without treatment. Five years later, the yellowish-white lesion and surrounding orange halo became funduscopically more evident than at the first visit (Fig. 1b).

**Fig. 1:**
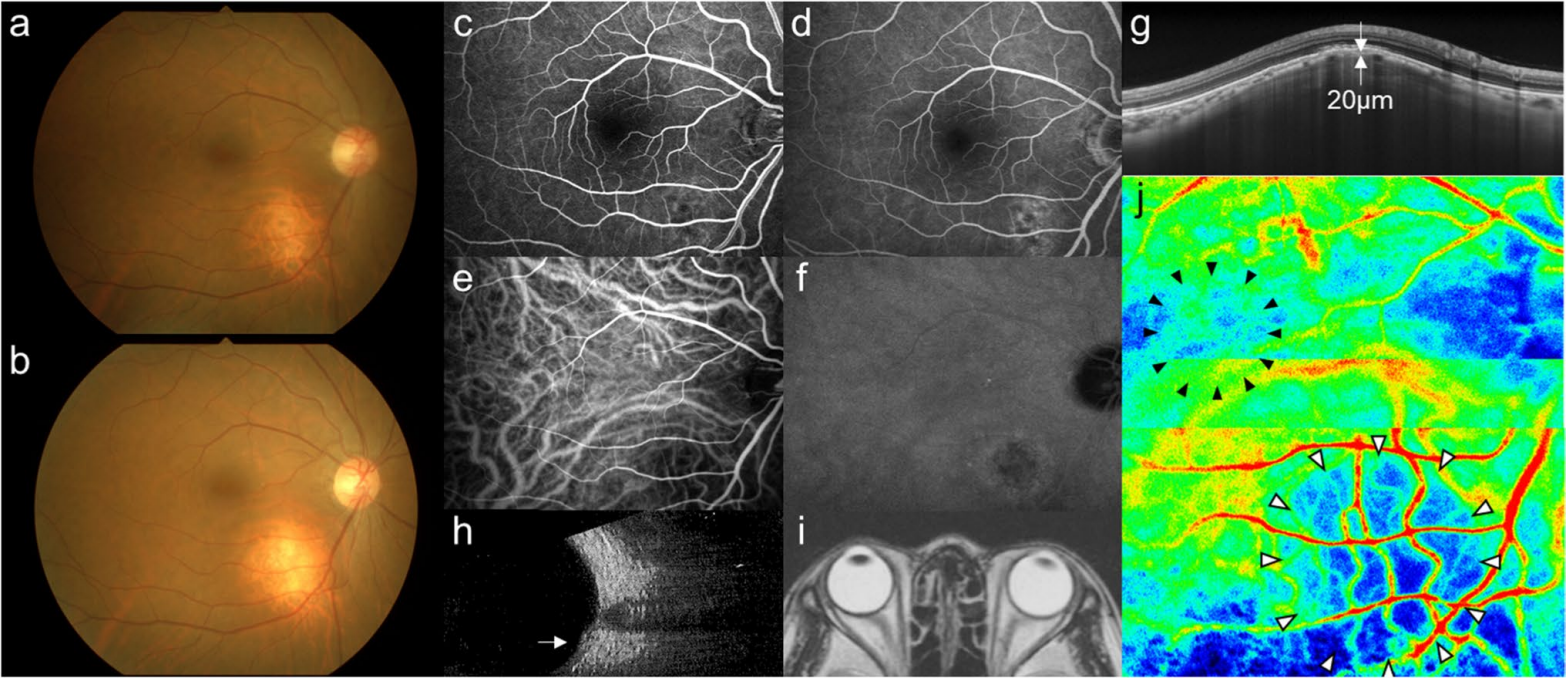
Images of the right eye in a patient (Case 1) with focal scleral nodule (FSN).** a** The fundus photograph at the initial visit showing an orange lesion with well-defined choroidal vessels less than the two-disc diameter at the inferonasal site of the macula. **b** The FSN lesion was yellowish-white, and the surrounding orange halo became evident 5 years later. **c** Early-phase fluorescein angiography (FA) shows scattered hypofluorescence and surrounding hyperfluorescence. **d** The hyperfluorescence turned to granular enhancement in the late phase of FA. **e** Early-phase indocyanine green angiography showing hypofluorescence at the lesion. **f** The hypofluorescence persisted with a new surrounding hyperfluorescence in the late phase. **g** Enhanced depth imaging optical coherence tomography showing the elevation of the sclera, with the overlying choroid thinned to 20 μm. **h** B-mode echography showing an elevated lesion (white arrow), but without acoustic shadow suggestive of calcification. **i** Contrast-enhanced MRI of the orbit shows no abnormal findings, including orbital tumors. **j** The laser speckle flowgraphy color map 1 year after the initial visit showing localized cooler color (white arrowheads) corresponding to the FSN lesion, indicating blood flow impairment

The LSFG color map of mean blur rate (MBR) showed localized cooler color at the lesion (white arrowheads, MBR = 4.6) than at the macula (black arrowheads, MBR = 7.7), indicating blood flow was disrupted in the FSN site (Fig. 1j).

Our second case was a 56-year-old woman who was referred to our clinic because of a yellowish-white lesion in her left macula. Past medical and family history was unremarkable. BCVA was 20/16 OS. The fundus and other findings were similar to those in Case 1 (Fig. 2a–i), but the elevation was closer to the macula and steeper. Late-phase FA showed hyperfluorescence across the fovea, indicating retinal pigment epithelial damage (Fig. 2d).

**Fig. 2:**
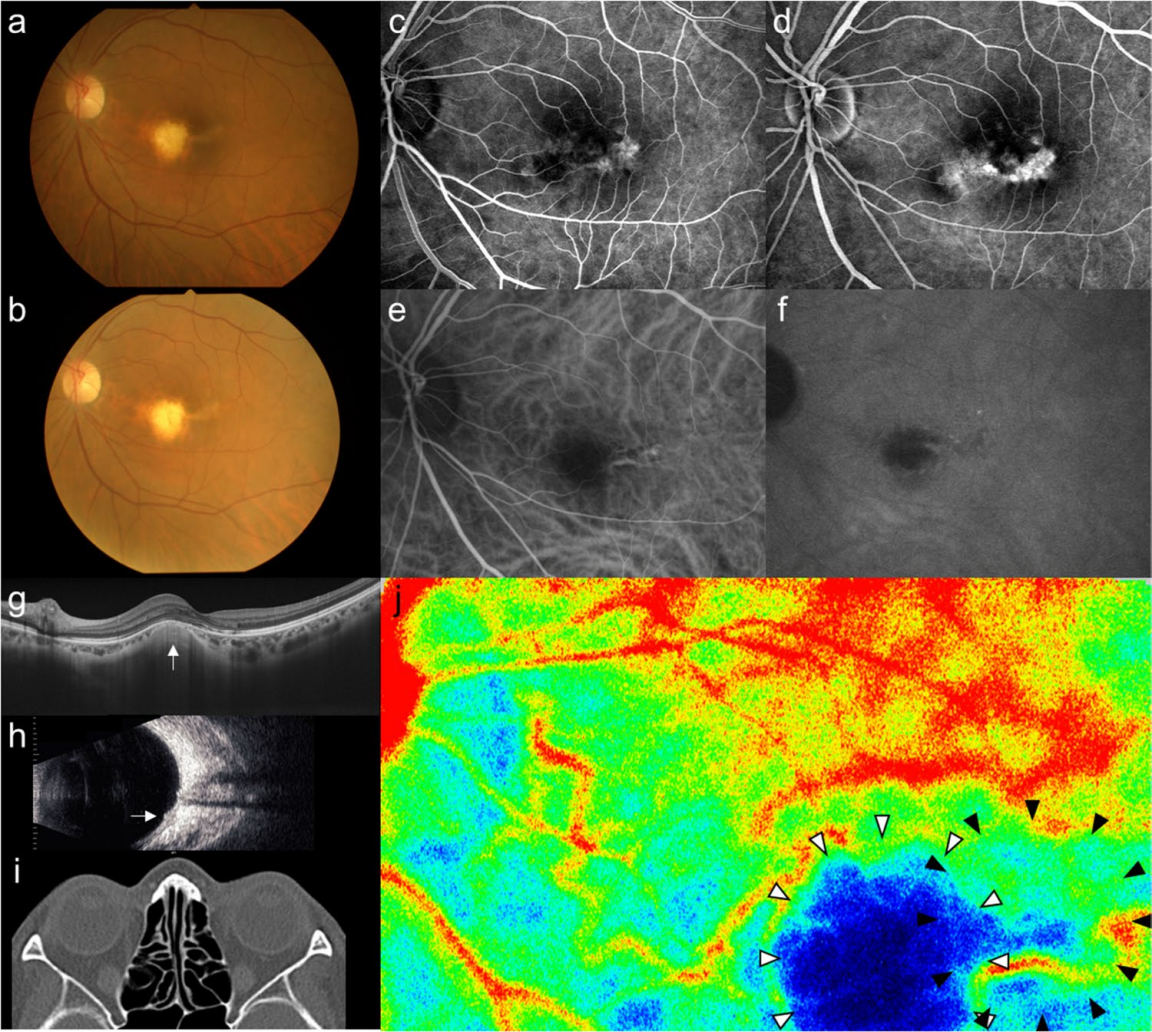
Images of the left eye in a patient (Case 2) with focal scleral nodule (FSN).** a** The fundus photograph at the initial visit showing a whitish-yellow lesion of about one-disc diameter with an orange boundary neighboring the fovea. **b** The lesion and surrounding orange halo became more evident in the fundus photograph 5 years later. **c** Early-phase fluorescein angiography (FA) showing window defects corresponding to retinal pigment epithelium atrophy around the whitish-yellow lesion. **d** The hyperfluorescence was enhanced in the late phase of FA. **e** Early-phase indocyanine green angiography showing low fluorescence at the lesion. **f** The hypofluorescence lesion persisted in the late phase. **g** Enhanced depth imaging optical coherence tomography showing the elevation of the sclera (white arrow), with the choroid compressed compared to the other parts of the choroid. **h** B-mode echography showing an elevated lesion (white arrow) with no acoustic shadow. **i** Contrast CT of the orbit showing no abnormal findings. **j** The laser speckle flowgraphy color map 3 months after the initial visit showing apparent solitary cooler color (white arrowheads) corresponding to the lesion

On LSFG, the lesion showed a cooler color (Fig. 2j white arrowheads, MBR = 3.3) than the macula (black arrowheads, MBR = 10.2), indicating blood flow reduction in the FSN site. Five years later, the lesion became more apparent than at the first visit (Fig. 2b).

Since the yellowish-white lesion in this disease was once considered an inflammatory disease of the choroid, Hong et al. named it unifocal helicoid choroiditis in 1997 [9], and Shields et al. solitary idiopathic choroiditis in 2002 [10]. However, recent EDI-OCT findings prompted Fung et al. to rename these lesions to FSN, reporting that these lesions originate from the sclera and not the choroid [1]. In both cases of this report, the cooler LSFG color showed a focal reduction in blood flow. It is noteworthy that the LSFG findings were strictly localized in the lesion. Conversely, in inflammatory diseases such as punctate inner choroidopathy, whose primary condition is choroiditis, LSFG shows reductions in blood flow beyond the visible area of involvement on clinical exam [7], whereas in FSN, reduction in blood flow on LSFG is noted only in the area of involvement on clinical exam. The localized blood flow reduction in LSFG is a finding supporting the pathophysiology of FSN, a primary scleral elevation, and the resultant decrease in choroidal blood flow due to mechanical compression of the choroid.

## Data Availability

Not applicable.
